# Directional MAC Approach for Wireless Body Area Networks

**DOI:** 10.3390/s110100771

**Published:** 2011-01-12

**Authors:** Md. Asdaque Hussain, Md. Nasre Alam, Kyung Sup Kwak

**Affiliations:** UWB-ITRC Center, Inha University, 253 Yongyun-dong, Nam-gu, Incheon, 402-751, Korea; E-Mails: m.asdaque@gmail.com (M.A.H.); nasarhi@gmail.com (M.N.A)

**Keywords:** wireless body area network (WBAN), MAC, multi-beam adaptive arrays (MBAA), sensor node, Slotted Aloha

## Abstract

Wireless Body Area Networks (WBANs) designed for medical, sports, and entertainment applications, have drawn the attention of academia and industry alike. A WBAN is a special purpose network, designed to operate autonomously to connect various medical sensors and appliances, located inside and/or outside of a human body. This network enables physicians to remotely monitor vital signs of patients and provide real time feedback for medical diagnosis and consultations. The WBAN system can offer two significant advantages: patient mobility due to their use of portable monitoring devices and a location independent monitoring facility. With its appealing dimensions, it brings about a new set of challenges, which we do not normally consider in such small sensor networks. It requires a scalable network in terms of heterogeneous data traffic, low power consumption of sensor nodes, integration in and around the body networking and coexistence. This work presents a medium access control protocol for WBAN which tries to overcome the aforementioned challenges. We consider the use of multiple beam adaptive arrays (MBAA) at BAN Coordinator (BAN_C) node. When used as a BAN_C, an MBAA can successfully receive two or more overlapping packets at the same time. Each beam captures a different packet by automatically pointing its pattern toward one packet while annulling other contending packets. This paper describes how an MBAA can be integrated into a single hope star topology as a BAN_C. Simulation results show the performance of our proposed protocol.

## 1. Introduction

According to aging estimates, there will be some 71 million elderly people in USA by the year 2030, more than twice the number in the year 2000 [1]. With this progressive rise of people living longer and the increasing segment of elderly in the population, there will be greater need for ways to monitor their medical status and keep them safe without forcing them to live at or near hospitals or healthcare facilities.

Recent advances in Wireless and Micro-Electro-Mechanical technologies and the proliferation of electronics gadgets in, on and around human body provide a unique opportunity for building the next generation of wireless BAN technology targeted at medical and consumer applications. WBAN is seen as the key technology that will provide a single unified solution for connectivity in and around the body, and which is intended to support a wide range of medical applications such as wellness monitoring, deep brain stimulation, electronic pills, and implanted drug delivery, as well as lifestyle applications including ambient intelligence (e.g., home, office, car), gaming, entertainment, and consumer electronics [2].

**Figure 1.**
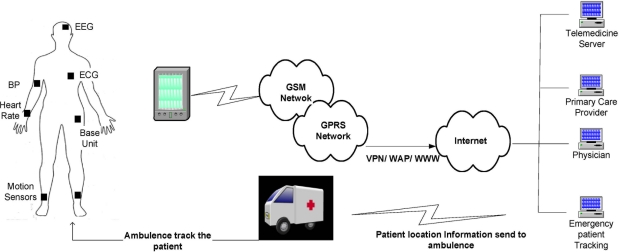
WBAN’s working scenario.

Figure 1 shows the general working scenario of a WBAN. Here all the data from a human body is collected and supplied to the appropriate destination using wireless communication. We are just concerned about the communication among the different sensors and BAN_C, which itself forms a small network. It has some distinctive properties which differentiate it from either a wireless sensor network or wireless personal area network. The close proximity of different sensors and BAN_C nodes to the body compels us to keep the electromagnetic pollution extremely low. The devices used (except BAN_C) are very small in their size (approx 1 cm^3^), which in turn put constraints on their energy consumption. Some of the devices are implanted inside the body with negligible option of renewing their energy source, so a long battery lifetime is needed (up to several years or even decades) [3]. Limitation on energy resources and available memory, consequently limits the computational power of such devices. Additionally, different sensors have different data rates and packet size. This small network may consist of numerous devices on a human body, which results into strong interferences. Electromagnetic waves are propagated through human body, so higher attenuation hampers the transmitted waves, before they reach their destination. It needs a simple and accurate propagation model as devices are quite heterogeneous in terms of data traffic, power consumption, delay and reliability. There is a large volume of ongoing work to develop the Medium Access Control (MAC) protocol for WBANs. Its distinctive property, which we discussed above, does not hold true for either Wireless Sensor Networks (WSNs) or Wireless Personnel Area Network (WPANs). This is why use of any of the set standard WSN or WPAN protocols does not meet the exact requirement of WBANs. In Table 1 we can see the IEEE draft WBAN specifications.

WSN has been the inspiration behind most of the designed WBAN protocols in the literature. The IEEE 802.15.4 standards for low rate WPAN have been analyzed extensively. Our proposal exploits the multibeam adaptive array technology with a slotted aloha scheme. We know that directional antenna have received attention for *ad hoc* network protocols, and recently, many directional antenna MAC protocols have been proposed for wireless *ad hoc* networks [4]. Use of directional antennas in communication offers many advantages such as increased gain, spatial reuse, reduced interference for signal detection, improved throughput, *etc.* Spatial reuse and simultaneous communication of a BAN coordinator with the different nodes is the key behind this proposal. In a medical care facility, in a critical patient, we are bound to provide a solution where more than one sensor can communicate with coordinator simultaneously. MAC design with the help of classic protocols like FDMA, TDMA, or IEEE 802.15.4 *etc.* cannot provide parallel communication between more than one sensor and a BAN coordinator. The use of multi beam or multi radio concept provides support to this kind of network. The use of MBAA in such small network is discussed in the related study part.

Our protocol is for WBAN, for which almost all of the works suggest a star topology incorporated with a coordinator at its center, hence we also considered this simplest topology with a maximum 25 sensor nodes in this work. The paper is further categorized in different sections. Section 2 has some related studies, Section 3 describes our MAC protocol, Section 4 deals with the simulation and results, and Section 5 concludes the work.

## 2. Related Studies

There are various works which have aimed to resolve the MAC requirement of WBAN. Its similarity with WSN and WPAN gave it a basis for comparison, so MAC protocols utilized by these two network types have been analyzed for use in WBANs, but their heterogeneous traffic and criticality related with their users (patients) makes them exclusive. In Table 2 we see some major differences between WSNs and WBANs [5].

Here we discuss some pros and cons of WSN-related MAC protocols, to verify whether they will smoothly match the vital specifications of WBANs. Existing MAC protocols, which are intended for WSNs can be broadly categorized as: (a) Low power listening based protocols; (b) Scheduled Contention based and (c) TDMA based protocols.

Low power listening based protocols like WiseMAC [6] and BMAC [7] are quite good for high traffic applications, but are not suited for the low duty cycle of in-body or on-body nodes. As far as STEM [8] is concerned, it seems good for periodic traffic, especially for low traffic applications. It is suitable for handling sporadic events due to a separate control sub channel, but not in the case of high traffic, which is one of the possibilities in WBANs.

Scheduled Contention based protocols such as SMAC [9] and TMAC [10] are good for high throughput applications. In TMAC early sleep problem causes the loss of synchronization, while SMAC does not suit a network where throughput is not a big concern. Both protocols with above limitation seem unfit for WBAN. DMAC [11] has better delay performance due to sleep schedules but this one is also loosely synchronized.

The TDMA based protocol FLAMA [12] is good in the case of low power applications and it is adaptable to high traffic applications. Both the LEACH [13] and HEED [14] protocols use a cluster head mechanism which switches as per requirement,, but in the case of WBANs, switching of cluster head is neither possible nor required.

In spite of these protocols there are different proposals exclusively for WBAN in IEEE 802.15.6. Table 3 is an overview of the proposed MAC by different parties of the TG6 working group [15]. Protocols like Heartbeat Driven MAC protocol (H-MAC) [16], Reservation Based Dynamic TDMA Protocol (DTDMA) [17], and Body MAC Protocol [18] are also worth discussing.

H-MAC is a TDMA based protocol, supported by an active synchronization recovery scheme where two resynchronization schemes are implemented. The proposal is based on a star topology and it exploits heartbeat rhythm information in order to synchronize the nodes and enhance the energy efficiency.

DTDMA is again a protocol based on the TDMA approach with a beacon enabled super frame structure. It is good for normal traffic. In this proposal time slots are assigned to the nodes, which has buffered some data to transmit and later these slots are released for other nodes.

The Body MAC protocol is also a TDMA based protocol having a superframe structure, identical to IEEE 802.15.4. But this superframe is divided into downlink and uplink frame, and the uplink frame is further subdivided into a contention access period and a contention free period.

One protocol similar to a low rate WPAN is the preamble based TDMA protocol [19]. With some ns-2 simulation results, it claims that to outperform the IEEE 802.15.4 and S-MAC in terms of energy efficiency.

We propose an idea which mixes the concept of multi beam adaptive array with a slotted aloha medium access protocol. This protocol is simple and effective to implement, if appropriate hardware is available. An important reasoning behind our proposal is to decrease the delay while accessing the BAN coordinator, enough time to sleep while no transmission is on the way, and simultaneous transmission by different nodes towards a BAN coordinator in case of urgency. Use of MBAA at the BAN coordinator provides the answer to the above problems pertinently. It is lacking in case of downlink transmission when some transmission is needed from BAN_C to sensor nodes. In the system model explanation section we describe the workings of MBAA with a slotted aloha scheme.

### 2.1. Relevance of MBAA in WBAN

As far as use of multi beam adaptive array in WBAN is concerned, several concerns come to mind. The first of these is that they are too big, impractical and expensive for WBANs. In response we recognize that this modern platform itself is an expensive tool and will be often used by the rich, so cost is not a big concern. Another concern is the impracticality, since frequency reuse is one of the crucial issues to cope up with. Ongoing work on antennas for enhanced spatial division multiplexing gives us confidence that sooner or later we are going to use multi beam antennas in all forms of wireless communication. Literature supports the notion of personal area networks using millimeter wave antennas as well as the multi beam antenna approach for gigabit wireless communication [20,21], which we could not have imagined in the 90s. In [22] Ramanathan reports that at 2.4 GHz, and the typical half wavelength element spacing, an eight element cylindrical array would have a radius of about 8 cm, making it quite unwieldy. As the operating frequencies continue to increase (as we know IEEE 802.11a uses 5 GHz), the antenna sizes will shrink. In the 5.8 GHz ISM band, the eight element cylindrical array will have a radius of only 3.3 cm, and at the 2.4 GHz ISM band a mere 0.8 cm. thus the future for the beam forming techniques for such small network also looks bright.

**Figure 2.**
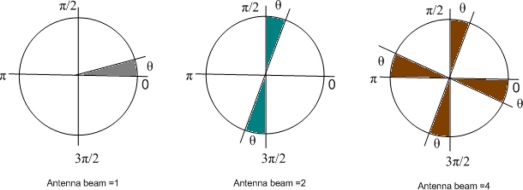
Beam pattern of antenna steering.

Our network has a star topology which couples the entire sensor node with the BAN Coordinator. It is generally presumed that the size of the coordinator will be somewhat equal to that of a PDA. Use of MBAA at coordinator will increase all the parametric performance. Logically this approach divides the human body into a number of arrays. In that case simultaneous transmission of data will reduce the critical nature of the network. Medical care in urgent situations creates circumstances where we need uninterrupted communication from more than one bio sensor devices. Use of multi beam or multi radio does not seem suitable for now, but a more careful examination opens up a number of possibilities. Figure 2 shows the pattern for the antenna beam steering.

## 3. System Model

In our approach of designing the MAC for WBAN, the slotted aloha multiple access method is merged with the MBAA. The human body is treated as a circular cell with its base station as the BAN coordinator, which is mounted with MBAA, while all the sensor nodes are using omni mode antennas. Sensors are fixed, so there is no mobility in the network but the whole network itself is mobile. In this fixed star topology network a slotted channel is shared by the sensor nodes in the uplink transmission (from node to coordinator). The BAN coordinator receives packets from the sensor nodes through N, (where N ≥ 1) different but spatially separated beams. Each beam is of θ radians, where θ ≤ 2π/N. If θ < 2π/N, these beams are non-overlapping, and the BAN coordinator is in receiving mode. The BAN area is also partially covered, leaving holes in the coverage, as we can see in the Figure 2, when antenna beam = 4. Angular width of the non illuminated holes will be (2π/N-θ). This scenario provides the room for the BAN coordinator for beam steering towards specific node without interference to others. When θ = 2π/N, BAN coordinator can be used to broadcast by switching on all the antenna elements N.

This small network notably has a very heterogeneous kind of traffic pattern with high risk urgent traffic situation. In case of any criticality to the patient it should be able to interact with BAN_C with very little delay. When sensors have nothing to transmit, they are in sleeping mode to save energy for longer lifetime. It has low traffic and low data rate. This network requires an efficient and simple MAC protocol to deal with its unique features. We reported some related studies in the previous section which elaborate different MAC and their capability to fulfill the requirements of WBAN. Due to the unique challenges posed by this small network each of those proposals has some drawbacks and cannot be used intact.

### 3.1. Slotted Aloha

Slotted aloha divides the time into equal slots, and the data packets are also kept of equal length. Packets are transmitted in fresh slots after their arrival. If more than one transmission takes place in a single time slot, packets are destroyed. There is a scenario where simultaneous transmission of several packets does not necessarily result in destruction of all the transmitted information. Using capture effect, if power of one of the received packet is sufficiently high, compared to the other packets involved in the collision, and then strongest packet can be correctly decoded while others will be lost [23].

### 3.2. Working of Our Model

**Figure 3.**
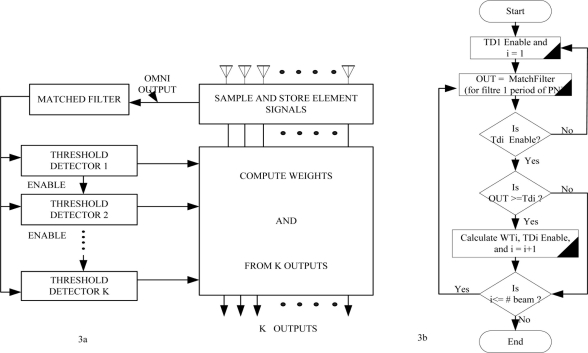
Flow chart and block diagram of MBAA and the signal acquisition signal processing [24].

Mixing MBAA with a slotted aloha makes it capable of receiving data from more than one sensor node in a single time slot, without the previously discussed capture effect. Every packet from different nodes transmitted in a slot is captured by a different beam of the MBAA simultaneously and it nullifies any other transmission which may occur in the same beam pattern. This technology allows BAN_C to receive more than one data packet in the same slot without any scheduling algorithm or reservation based protocol. Figure 3 show the data transmission flow.

In our model we assume that in a human body various kind of biosensors are implanted inbody or onbody as per the different requirements. These sensor nodes send their data to the BAN coordinator. When some sensor node sends data to the BAN_C, it is demodulated and errors are checked with the use of error detection code. If a packet is error free it is successfully accepted, otherwise it is discarded.

When a packet is successfully received an ACK is sent by the BAN_C to the sensor. This ACK is always sent on another frequency so that BAN_C and sensor nodes can transmit and receive simultaneously.

The main obstacle in the use of MBAA in a WBAN network is the acquisition of packets, i.e. the problem of locking each beam onto a different packet, while annulling all other packets in the slot. For this we use following technique for acquisition: in each of the slot we add a preamble. This preamble keeps three period of a known pseudo noise sequence [24]. Slot width is increased as compared to the data packet with uncertain time period. Within this time period, packet transmission is randomized so that each packet reaches to BAN_C at a slightly different time.

Packets are then acquired as follows, suppose at first only one beam is to be formed. The goal is to point the beam toward the first packet to arrive in each slot with nulls on any other packet arriving in that slot. For packet acquisition, a single array element with an omni directional pattern will be used as the receiving antenna, so any packet can access the system. When a second packet arrives in same slot the first packet will be received successfully, on the condition that the second packet should reach there with at least one bit delay [24]. If condition is not met, the array will lose both the packets. In Figure 3(a), we see that if multiple packets are coming in one slot from different beams then packets with a slight delay are acknowledged successfully. Multiple packets are received simultaneously with the use of the packet acquisition technique. A separate threshold detector and weight calculation is used by each adaptive array beam. Arrival of a packet triggers the threshold detector for the next incoming packet. For example if the first packet arrives, it triggers threshold detector one (TD1), when TD1 is triggered it will enable TD2 so that it can receive the next packet if it arrives (TD2 will not operate until after TD1 has been triggered). The same approach is taken successively for new packet arrival until the maximum number of beam patterns is reached. Figure 3 has the block diagram of a multi-beam adaptive array and the acquisition signal processing. The packet acquisition technique is elaborated in detail in [24].

## 4. Simulation Section

For simulation of this work we have used the OPNET modeler wireless suite to characterize the network performance [25]. This tool facilitates antenna modeling in its antenna pattern editor and its editor supports the creation of the arbitrary 3D gain patterns. The beam can be pointed at desired points in three dimensions, and the energy received at every node is computed automatically by the OPNET kernel procedures. With addition of a child process in a contributed slotted aloha model we modeled our scenario [26].

**Figure 5.**
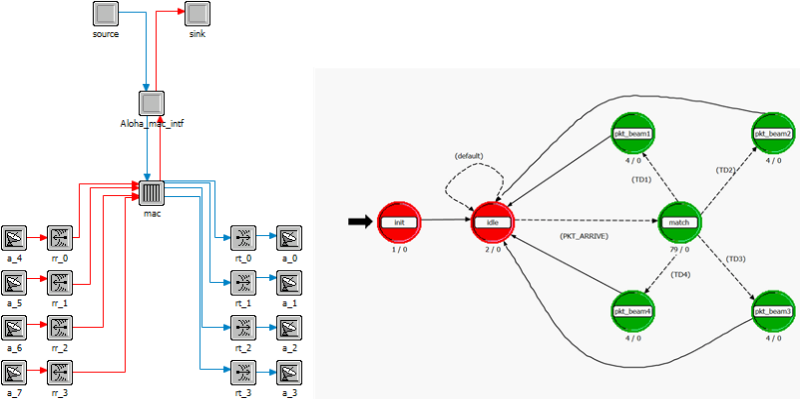
Node and process model for MBAA implementation.

Figure 5 is a node model and child process model used for addition of MBAA functionality. In the node model we have attached four antenna patterns; each of them has 30 degree directionality and 2 dB gain. They are programmed to track the node position, and point toward the aimed transmission. The MAC processor of the node model contains the aloha process model, and here the child process (in Figure 5) is called. When an interrupt occurs through the stream, the control goes to match state. If the packet comes from the higher layer, it is sent to the physical layer for transmission.

Otherwise the packet is received from the physical layer then the match filter algorithm is in process to calculate the weight [see Figure 3(b)]. As per the calculated weight packet, acquisition takes place and goes to the related states (e.g., pkt-beam1, pkt-beam2 *etc.*). The important simulation parameters are listed in Table 4.

**Figure 6.**
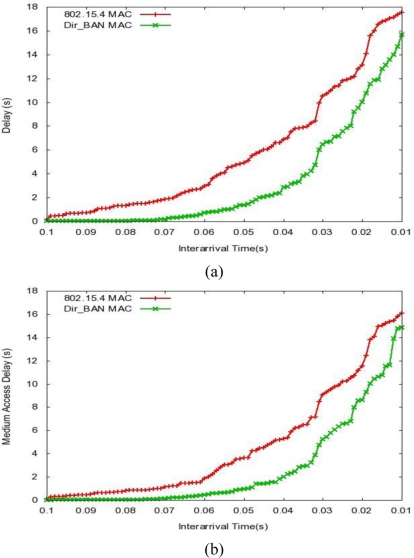
**(a)** End to End Delay *vs.* Inter arrival Time. **(b)** Medium Access Delay *vs.* Inter arrival Time.

**Figure 7.**
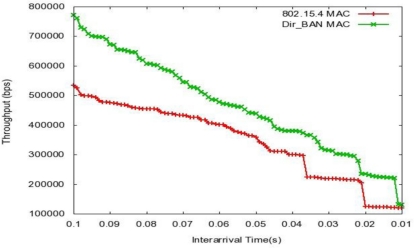
Throughput *vs.* Inter arrival Time.

Simulations results of the IEEE 802.15.4 and Dir_BAN (MBAA with slotted aloha) are presented under varying number of inter arrival times to the network. The performance metrics concerned in this research work are mean delay, MAC delay, and throughput. Figure 6 represents the end to end delay of all the packets received by the MACs of all nodes in the network and forwarded to the higher layer. Figure 7 represents the total of queuing and contention delays of the data frames transmitted by all MAC. For each frame, this delay is calculated as the duration from the time when it is inserted into the transmission queue, which is arrival time for higher layer data packets and creation time for all other frames types, until the time when the frame is sent to the physical layer for the first time. In Figures 6 and 7 we see that decreasing inter arrival time is (frequency of data arrival is increasing) causing more data frames generation, which causes more delay and it goes up exponentially with the decrease in inter arrival time. The proposed approach is effective in reducing both the delays.

Figure 7 is the representation of the total number of bits (in bps) forwarded from the MAC to higher the layer, *i.e.*, throughput. Throughput also surpasses that of the classical IEEE 802.15.4 protocol.

## 5. Conclusions

This paper introduces a new approach for a WBAN MAC protocol. The simulation results of the proposed MAC with MBAA are presented and compared with those of a regular IEEE 802.15.4 omni directional mode. It also aims to find a way of applying smart antenna technology the smallest networks. By just mounting an adaptive array on the BAN coordinator we are capable of enhancing the performance. We have discussed the relevance of the use of MBAA in WBAN and packet acquisition technique in case of simultaneous transmission. In future work we plan to include prioritization of different sensor nodes with unique inter-arrival time, packet size and data rate.

## Figures and Tables

**Figure 4.**
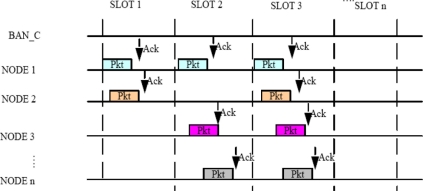
Diagram of transmission between BAN_C and nodes acquisition signal processing.

**Table 1.** IEEE WBAN specification.

Distance	2 m Standard 5 m special use
Network Density	2–4 nets/m^2^
Network Size	Max: 100 devices/network
Power Consumption	∼1mW/Mbps
Startup Time	<100 μs or <10% of Tx slot
Latency	10 ms
Network setup time	<1 s (Per device setup time excludes network initialization)
Effective sleep modes	
Operation in global, license-exempt band	
Peer to Peer, and Point to Multi-point communication	
Future proof	Upgradeable, scaleable, backwards compatible
Quality of Service & Guaranteed Bandwidth	
Concurrent availability of asynchronous and isochronous channels	
Very Low, Low, and High duty cycle modes	Allows device driven degradation of services

**Table 2.** Difference between WSNs and WBANs.

**WSNs**	**WBANs**
Cover the environment	Cover the human body
Large number of nodes	Fewer sensor nodes
Multiple dedicated sensors	Single multitasking sensors
Lower accuracy	Robust and accurate
Resistant to noise	Predictable environment
Failure reversible	Failure irreversible
Fixed structure	Variable structure
Low level security	High security
Accessible power supply	Inaccessible power source
High power demand	Lower power availability
Solar, wind power	Thermal, piezoelectric energy
Replaceable/disposable	Biodegradable
No biocompatibility needed	Biocompatible
Wireless solutions available	Lower power wireless
Data loss less of an issue	Sensitive to data loss

**Table 3.** MAC proposals for IEEE 802.15.6.

**MAC Proposal**	**Type**	**Brief Description**
MedWin	Beacon	Star topology, time partitioning, beacon, channel migration, security
NICT	Beacon/Non Beacon	Super frame, TDMA based, non-beacon mode, MICS for wakeup
IMEC	Beacon	Dual duty cycling, flexible & power efficient, enhanced slotted Aloha with QoS, wakeup receiver, priority-guaranteed
YNU	Not mentioned (Cluster based communication)	Protocol considering SAR or thermal influence to a body by switching cluster
Samsung	Polling	Piconet co-existence, network management and security, poll based access and Single MAC concept
Inha	Beacon	Wakeup by Traffic Patterns and Radio, Super frame, MAC frame structure, Security, Multiple PHY support, Bridging Function
Fujitsu	Beacon	Signaling covering emergency, reliability, congestion and stability and wake up concept
CSEM	Preamble based WiseMAC-HA	WiseMAC based proposal ( WiseMAC-HA)

**Table 4.** Simulation parameters.

Network Area	8 × 5 feet
Topology	Star
Number of nodes	25
BAN Coordinator	Directional Mode
Sensor nodes	Omni directional mode
Directional gain	2 dB
Packet Inter-arrival time	Exponential (0.1 to 0.01)
Packet size	1,024 bits (mean outcome)
Simulation Time	600 s
Number of Seeds	128
Frequency Band	2.4 GHz
Data Rate	1,024 bps
